# Lubricin: A Principal Modulator of the Psychoneuroendocrine - Osteoimmune Interactome - Implications for Novel Treatments of Osteoarthritic Pathologies

**DOI:** 10.6026/97320630013343

**Published:** 2017-10-31

**Authors:** Allen Khakshooy, Nicole Balenton, Francesco Chiappelli

**Affiliations:** 1 Laboratory of Human Psychoneuroendocrine-Osteoimmunology; School of Dentistry, UCLA Center for the Health Sciences, Los Angeles, CA 90095-1668;; 2 Evidence-Based Decision Practice-Based Research Network, DGSO, Los Angeles, CA 91403; 3 Department of the Health Sciences, CSUN, Northridge, CA 91330; 4 Rappaport Faculty of Medicine, Technion-Israel Institute of Technology, Haifa, Israel 3109601

**Keywords:** Lubricin, interactome, osteoimmunology, psychoneuroendocrine-osteoimmunology, osteoarthritis, CRISPR

## Abstract

Lubricin is a synovial glycoprotein that contributes to joint lubrication. We propose the hypothesis that lubricin is a key modulator of the psychoneuroendocrine-osteoimmune interactome, with important clinical relevance for osteoarthritic pathologies. We consider a variety of neuroendocrine-immune factors, including inflammatory cytokines and chemokines that may contribute to the modulation of lubricin in rheumatic complications. Based on our preliminary immunocytochemistry and fractal analysis data, and in the context of translational research of modern healthcare, we propose that molecular lubricin gene expression modification by means of the novel CRISPR/Cas system be considered for osteoarthritic therapies.

## Background

Osteoimmunology is a relatively new interdisciplinary domain of biological research, which focuses on the constellation of interactive processes that cross-modulate cellular immune surveillance and bone metabolism. The field originated as the elucidation of the immune regulation of osteoclasts. It now encompasses a wider scope that extends from molecular and cellular interactions, to genomic and interactomic operational events [1].

The term interactome refers to the set of molecular and cellular interactions, including gene interactions, gene product interactions, molecule-protein interactions and the like, that characterize an organism, or an organ within it. Interactomes are more than simply molecular or physiological networks-they display feedback properties that are essential for the organism's survival. Interactomics is a novel discipline at the intersection of bioinformatics and biology that investigates the fundamental mechanistic principles of interactomic feedback and their consequences in health and disease. One important interactomic set of relationships is that which coordinates the intertwined cross-regulatory feedback between the psycho-neuroendocrine and immune systems, which we previously discussed in the context of patients with HIV/AIDS afflicted with the immune reconstitution inflammatory syndrome (IRIS) [2] or with Alzheimer's disease [3]. Taken together, these lines of investigations have led us to propose a new field of interactomic research, which we labeled psychoneuroendocrineosteoimmunology [4], that directly pertains to the field of osteoarthritic pathologies [5, 6].

Related research has established that the 340-350 kDa protein lubricin, often referred to as proteoglycan-4 (PRG4), is present in synovial fluid, on the superficial layer of articular cartilage, and plays an important role in joint lubrication and synovial homeostasis. This superficial zone protein is synthesized by chondrocytes located at the surface of articular cartilage and by fibroblastoid synovial lining cells. Lubricin's glycosylated region - the mucin domain - renders it hydrophilic, which permits its interaction with galectin-3 and promotes its lubricating property. Its remaining non-glycosylated regions interact with cartilage proteins, and aid in lubricin's boundary lubricating ability [7, 8, 9]. A large family of enzymes, immune factors, including cytokines and chemokines, peptides, and other modulators of physiological processes contribute to regulate lubricin biosynthesis. The importance of these interactomic sets of physiologic feedback is exemplified by the profound pathophysiological phenotypes of osteoarthritis observed in animal models and human patients. Indeed, recent findings implicate inflammatory factors in the attenuation of lubricin-mediated synovial lubrication in osteoarthritic pathologies [10].

Taken together with observations reported in a systematic review of increased levels of pro-inflammatory cytokines in the synovial fluid of osteoarthritic joints [11], as well as our own data [5, 6], we propose the hypothesis that lubricin is a key modulator of the psychoneuroendocrine-osteoimmune interactome. Our corollary hypothesis states that the regulation of synovial lubricin levels by TH17 cytokines has clinically relevant implications for novel treatments of osteoarthritic pathologies, such as CRISPR/Cas9- mediated genomic modification.

## Methodology

Our experiments toward testing this hypothesis involved primary cultures of plastic-adherent human synovial fibroblasts (human fibroblast-like synoviocytes, HFLS, Adult 408-05A, Aldrich) maintained under exponential growth condition in serum-free SCMF001 culture medium (Millipore) at 37°C in a 5% CO2 atmosphere. Stock cultures were maintained in 75cm2 plastic flasks (Falcon), and subcultured by standard trypsinization (0.1% trypsin in Dulbecco's phosphate buffered saline, PBS, 37°C, 5 min) into 24-well plastic plates (Falcon) containing a tissue culture-treated sterilized glass coverslip at 105, 2.4X105 or 5X105 cells/well depending on the experiment, 24 h before testing. Cell density and morphology was verified by phase contrast microscopy. Cell counts were obtained by standard hemacytometer independently by two standardized cell culturists.

In vitro modulation of lubricin expression can tested under experimental conditions by supplementing the growth medium during the last 24 h of the experimental culture period with micro-filtered spent medium from related cultures, including myeloid (THP-1) and lymphoid cell lines (Jurkat) maintained in serum-free AIM-V medium (Gibco) and activated as needed [12], and other cell culture growth supplements, as required by the specific experimental conditions. The effect of the immune factors of interest (i.e., cytokines, chemokines) can be blocked by specific monoclonal antibodies (Becton Dickinson) added to the experimental culture medium. Confirmatory experiments can test the addition of micromol concentrations of the recombinant form of the identified human immune factors (Becton Dickinson).

The experimental outcome in our model is the immunoreactive form of lubricin, as detected by immunocytochemistry [13] using a polyvalent rabbit anti-human lubricin antibody (MABT401, Millipore) in a standard immunocytochemistry experimental protocol [13] optimized for lubricin detection. In brief, cultures were fixed in 3.7% formaldehyde in PBS (room temperature, 10 min) with or without simultaneous permeabilization (0.1% triton X-100 in the formalin solution). Following copious washes in PBS (room temperature, 30 min), the cultures were incubated in the antibody solution at diverse dilutions (1/50-1/500, depending on the experimental conditions, PBS) (60-120 min, room temperature to overnight, 4°C). Following a second set of copious washes (room temperature, 30 min), the cultures were incubated in a 1/250 PBS dilution of biotin-conjugated goat anti-rabbit polyvalent antibody (Sigma) (60 min, room temperature), immediately followed by incubation with avidin-biotin complex (ABC) kit (Vectastain PK-4002, Vector Laboratories) as per the protocol recommended by the manufacturer. Horseradish peroxidase precipitable color development was obtained with diaminobenzidine (DAB) substrate and H2O2 as co-factor with a commercially available color-development kit (BioRad). The coverslips were mounted on glass slides in 10% glycerol for preservation, and microphotography (Nikon, with or without phase contrast, (20X objective X 10X eyepiece, and as need 100X oil immersion X 10X eyepiece).

**Figure 1:**
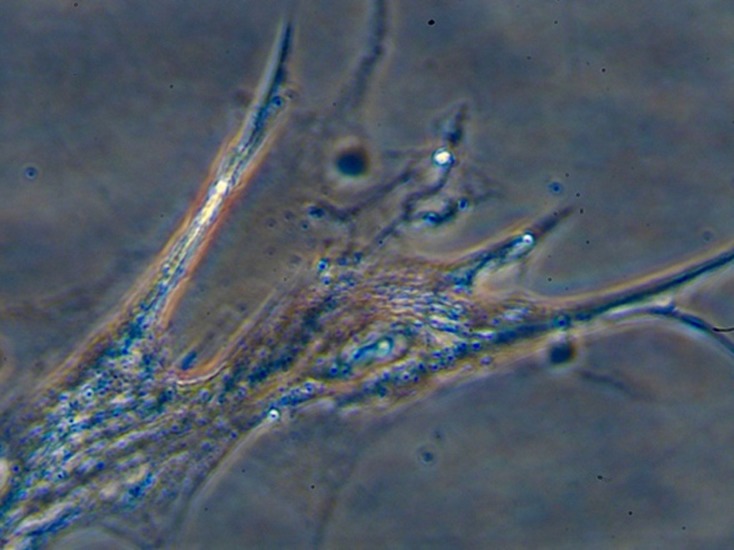
Lubricin immunoreactivity in a mixed culture of human synovial fibroblasts. This representative slide shows that lubricin appears to be expressed preferentially in smaller elongated fibroblastoid cells in the G1 phase of the cell cycle, as opposed to larger more spread-out cells, a morphology typically observed in fibroblasts in the G2 phase of the cycle. The figure also shows an apparent evolution in immunoreactive lubricin from an earlier stage of production, when it presents in a more diffuse form in the cytoplasm, presumably immediately following mRNA translation, and secondarily when lubricin appears in long filamentous immunoreactivity along the inner edges of the cell membrane. It is possible and even probable that the translocation of post-translational lubricin is directed by, and occurs via the cytoskeletal network. Taken together our observations suggest that cyclins and cyclin-dependent kinases, which together drive and regulate the progress of the cell through the gates of the cell cycle, on the one hand and cytoskeletal proteins, including tubulin, the principal constituent of cellular microtubules, actin, the component of microfilaments, and laminin in intermediate filaments, on the other hand play a concerted regulatory role for in the modulatory effects of lubricin in the psychoneuroendocrine-osteoimmune interactome. Our observations to date do not suggest ubiquitin-mediated proteolysis and lubricin degradation during the process of lubricin production and translocation.

Microphotographs in gray-scale (Figure 1) were quantified by fractal analysis as described [13], using the box-counting method with the Fractalyse software (Fractl-yse, fractalyse.org). Values of total cell dimensionality (d) varied over a range of 1.25-1.75, depending on the stage of the cell cycle - G2 cells being typically considerably larger than G1 or S cells. The anti-lubricin reactive domain within the cytoplasmic compartment was analyzed for fractal dimensionality, and the proportion of lubricin-stained dimension vs. total cell dimension was computed, tabulated (Table 1), and analyzed statistically using the MedCalc biostatistics software (medcalc.org).

## Discussion

Our studies to date were designed to quantify the expression of lubricin in human synovial fibroblasts under certain controlled experimental conditions by fractal analysis (Table 1) [13]. Localization of lubricin immunoreactivity varied depending upon the stage of the cell cycle, and the cytoskeletal microstructure of the cell (Figure 1). The translocation and progressive segregation of lubricin from the cytoplasm to the plasma membrane, seemingly occurs via the cytoskeleton in preparation of G2 cells undergoing cell division. We observe no ubiquitin-mediated proteolysis and lubricin degradation during the process of lubricin production and translocation. It is possible and even probable that cyclins and cyclin-dependent kinases, which together drive and regulate the progress of the cell through the gates of the cell cycle, on the one hand and cytoskeletal proteins, including tubulin, the principal constituent of cellular microtubules, actin, the component of microfilaments, and laminin in intermediate filaments, on the other hand play a concerted regulatory role for in the modulatory effects of lubricin in the psychoneuroendocrine-osteoimmune interactome.

Taken together, these preliminary experiments validate the in vitro model of human synovial fibroblasts to define and characterize the modulatory interactome of lubricin expression. Future studies need now to test specifically the most likely inflammatory immune factors that might be involved in the regulation of lubricin expression, based on the best available clinical literature on osteoarthritic pathologies.

The best evidence base to date, derived from the studies by Szychlinska and collaborators [10], de Silva and collaborators [11], and others, including data from our laboratory [5, 6], suggests that pro-inflammatory cytokines produced by myeloid populations, including interleukin (IL)-1b, IL-6 and tumor necrosis factor (TNF)-a will be found to play an important role in this context. Expectations are also that cytokines of the TH17 and TH9 profile may also play an important role in the regulatory modulation of lubricin expression. These cytokines are produced by lymphoid sub-populations of activated mature naive and memory T cells, known to be involved in sustained inflammation such as occurs in osteoarthritic joints.

The experimental model we validated here will provide a useful protocol by which: [1] a complete profile of cytokines produced by activated myeloid or lymphoid populations can be tested in vitro, [2], the selective use of specific monoclonal antibodies can be tested for their capacity to abrogate the outcomes observed in the in vitro model, and [3], the nature of the modulatory cytokine can be verified by means of its purified form added to the in vitro model under controlled conditions of concentration and time course. Pre-clinical and clinical studies should follow this in vitro characterization of the model using adaptive cluster randomized stepped wedge blinded controlled trial (CRSWBCT) designs, whose statistical power we have discussed previously in the context of testing novel clinical interventions for patients with osteoarthritic pathologies of the temporomandibular joint (TMJ) [14, 15]. Ultimately, these trials will specifically test the hypothesis and corollary hypothesis proposed here to define and characterize novel therapies across a variety of patients with osteoarthritic pathologies.

Current trends in molecular medicine and clinical studies of interactome-based interventions point to genomic manipulations by means of the CRISPR/Cas protocol. In brief, the clustered regularly interspaced short palindromic repeats (CRISPR) - CRISPR-associated proteins (Cas) system is currently the most promising genome engineering method that enables controlled modifications in selected genome sequences for basic research purposes, along with the development of new and improved therapeutic or biotechnological interventions. In fact, CRISPR/Cas systems show considerable diversity that is conferred by the Cas protein itself. Two classes of CRISPR/Cas systems are distinguished based on either the system having several Cas proteins (class I) or a single Cas protein (class II). Both class I and class II CRISPR/Cas are further organized in subtypes [16, 17].

Mechanistically, CRISPR/Cas proceeds in a stepwise manner, in which the bacterium responds to a foreign DNA during the acquisition phase. Secondarily, Cas1 and Cas2 nucleases cleave the foreign DNA, and the resulting fragments are inserted within a CRISPR locus in the bacterial genome between palindromic repeats. Transcription of the CRISPR locus results in a precursor RNA (CRISPR-RNA, crRNA) that hybridizes with the transactivating crRNA (tracrRNA). TracrRNA is small RNA whose sequence is complementary to the palindromic repeats, and allows Cas6 (aka, ribonuclease III) to cleave crRNA into mature fragments. Ultimately, the Cas/crRNA/tracrRNA complex (i.e., crRNP) is formed, which scans the bacterial genome to identify and cleave the inserted viral DNA [16, 17].

In brief, CRISPR/Cas confers adaptive immunity to a bacterium via a mechanism that requires the activation of the Cas nuclease during its association with the unusual genome structure consisting of palindromic repeats of about 30 base pairs (bp) separated by spacer segments of about 36 bp, referred to as CRISPR, into which viral DNA fragments are interspersed. In fact, over time, the acquisition of several viral DNA fragments by the CRISPR locus via the Cas pathway provides the bacterium with resistance to multiple viral DNAs. From that viewpoint, it has become evident that CRISPR/Cas9 system efficiently serves to reprogram bacterial immunity-thus, establishing the CRISPR/Cas9 system as a reliable and effective means of genome editing in eukaryotes [16, 17, 18].

CRISPR/Cas9 technology has revolutionized the field of genome editing in medicine. Novel molecular clinical interventions are being developed and tested that is based on the CRISPR/Cas9 system to add, delete, or modify the genomic material in patient populations afflicted with certain interactomic conditions. Nonetheless, the applications of the CRISPR/Cas9 molecular technology to clinically therapeutic interventions are still cutting edge. The caveat that remains is a vector capable of efficiently, specifically, and safely conveying the CRISPR/Cas9 components to the target cell or tissue must be developed, tested, and validated in Phase I and Phase II clinical trials, before it can be safely tested on patients [17, 18].

## Conclusion

In brief, the CRISPR/Cas9 system is a versatile molecular platform for introducing targeted genome modifications into mammalian cells. To ensure safe and efficient delivery into relevant cell types, adeno-associated virus (AAV) vectors are an efficient class of gene-delivery vehicles that safely infect dividing and non-dividing eukaryotic cells, and serve as a highly effective donor template for homology-directed repair. Used together, CRISPR/Cas9 and AAV technologies can accelerate both basic research and clinical applications of genome engineering [18].

Case in point, the AAV-mediated Atp6v1c1 knockdown gene therapy has been shown to effectively treat bone erosion and inflammatory bone damage caused by periodontitis in a mouse model [19]. The broad array of rheumatic diseases ranges from rare monogenic auto-inflammatory diseases to complex polygenic autoimmune diseases. In association with AAV vectors to enhance reliability and effectiveness, correcting abnormalities in the genome using CRISPR/Cas9 should undoubtedly improve not only our knowledge of molecular models of therapy, but also the benefit toward patients [20]. A promising human chondroitin sulfate proteoglycan-4 (i.e., lubricin) CRISPR/Cas9 guide RNA is now being developed and tested (genscript.com/gRNAdetail/ 1464/CSPG4-CRISPR-guide-RNA.html).

The next step will build on this paradigm and develop a CRISPR/Cas9 - Atp6v1c1 - guide RNA system for treating osteoarthritic inflammation, for the direct purpose of blunting the psychoneuroendocrine-osteoimmune interactomic effect on lubricin. This information will help develop new and improved molecular-based patient-centered therapeutic model of clinical intervention paradigms for osteoarthritic pathologies.

## Figures and Tables

**Table 1:** Lubricin immunoreactivity fractal dimension expressed as percent of total cell fractal dimension in a representative in vitro cultured G1 human synovial fibroblast.

Fractal Dimension (whole cell)	Fractal Dimension (lubricin immunoreactivity)	Percent Fractal Dimension (%)
1.579	1.763	(1.579/1.763)*100 = 89.56%
